# An ultrasensitive angular interrogation metasurface sensor based on the TE mode surface lattice resonance

**DOI:** 10.1038/s41378-024-00848-5

**Published:** 2025-01-08

**Authors:** Liye Li, Wengang Wu

**Affiliations:** 1 National Key Laboratory of Advanced Micro and Nano Manufacture Technology, Beijing, 100871 PR China; 2 School of Integrated Circuits, Peking University, Beijing, 100871 PR China; 3 Beijing Advanced Innovation Center for Integrated Circuits, Beijing, 100871 PR China; 4 Frontiers Science Center for Nano-optoelectronics, Peking University, Beijing, 100871 PR China

**Keywords:** Nanophotonics and plasmonics, Biosensors, Optical sensors

## Abstract

The localized surface plasmon resonance metasurface is a research hotspot in the sensing field since it can enhance the light-matter interaction in the nanoscale, but the wavelength sensitivity is far from comparable with that of prism-coupled surface plasmon polariton (SPP). Herein, we propose and demonstrate an ultrasensitive angular interrogation sensor based on the transverse electric mode surface lattice resonance (SLR) mechanism in an all-metal metasurface. In theory, we derive the sensitivity function in detail and emphasize the refraction effect at the air-solution interface, which influences the SLR position and improves the sensitivity performance greatly in the wide-angle. In the measurement, a broadband light source substitutes the single-wavelength laser generally used in traditional angular sensing, and the measured SLR wavelength of broadband illuminant at normal incidence is defined as the single wavelength, avoiding the sensitivity loss from the large angle. The experimental sensitivity can reach 4304.35°/RIU, promoting an order of magnitude compared to those of SPP-sensors. This research provides a novel theory as well as the corresponding crucial approach to achieving ultrasensitive angular sensing.

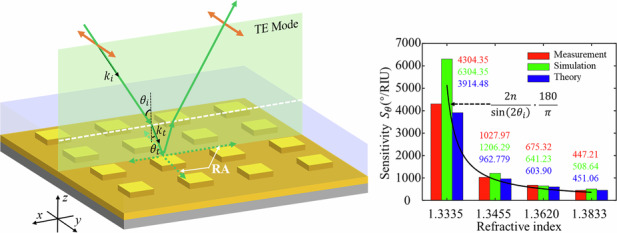

## Introduction

The localized surface plasmon resonance (LSPR) is a collective oscillation of electrons on the surface of metal particles with subwavelength sizes^1–3^. As a kind of planar optical component composed of arranged arrays of patterned nanostructures, the LSPR metasurface can support multiplex LSPR-based optical responses, such as absorption, holographic imaging, and polarization conversion. In particular, it can act as a nanoscale sensing platform to enhance the light-matter interaction and detect environmental reflective index (RI) change in real-time with rapid, accurate, and in-situ^4–6^ properties, which has attracted extensive attention in recent years^7,8^. However, compared with the commercial prism-coupled surface plasmon polariton (SPP) sensors^9–12^ the LSPR metasurface sensors have some shortcomings in performance, including higher Ohmic losses, lower quality factors, and smaller wavelength-interrogation sensitivities. Notably, the proposed sensitivity is defined as the resonance wavelength redshift normalized by a change in the RI^13^which is the most common sensitivity evaluation criteria. Although numerous research can restrain losses to improve partial LSPR sensing behaviors based on Fano resonance^14^, electromagnetically induced transparency^15^, quasi bound states in the continuum^16^, and other mechanisms^17–20^, the wavelength sensitivity is still about three orders of magnitude smaller than that of SPP and cannot be promoted effectively. In essence, the sensitivity value is mostly limited by the finite metasurface lattice length^21^, which is required to be near the working waveband in the design for excellent optical effects^22^.

In addition to analyzing wavelength redshift, the resonance angle is also a significant freedom to detect the RI change in the case of single-wavelength excitation, because the angle needs to match the increased RI to keep resonance at the incident single wavelength still^23–25^, with higher detection resolution and stability. Similarly, the definition of angular interrogation sensitivity is the angle variation change under unit RI change^26^. At present, almost all research about angular sensing depends on the SPP mechanism, excited by a laser with transverse magnetic (TM) mode. In theory, Homola J. et al. demonstrated that the SPP sensor based on a grating (one-dimensional metasurface) has much less wavelength sensitivity but similar angular sensitivity in contrast with the SPP from the prism couple^27^, laying a theoretical foundation for metasurface-based angular interrogation sensing. In numerical simulation, Hui W. et al. coated graphene oxide on the surface of a trapezoidal grating sensor, where the two-dimensional materials can improve light absorption and under-tested material sorption to make the angular sensitivity reach 350°/RIU^28^. In the experiment, Saeid N. et al. fabricated a rectangular Ag-MgF_2_ grating with an angular sensitivity of 85.61°/RIU and analyzed the influence of period length, laser wavelength, and meta-atom thickness for the SPP angle^29^. Despite many achievements, this research field has a remarkable problem which is an only single guiding theory (SPP)^30–32^^.^ Although there are many design methods in the metasurface structure, such as introducing composite metal layers and two-dimensional materials^33^, they do not break the limitation of SPP essentially and cannot promote the angle interrogation sensitivity effectively. There is some research based on other resonance modes, such as guided-mode resonance^34^, the sensing performance and practicality are lower than those of SPP. Besides, the above studies have ignored the refraction phenomenon on the interface between the air and the solution under-tested, without enough accuracy. The incident single wavelength is limit restricted by the laser source and requires a large angle generally, raising the measurement difficulty.

In this paper, we propose and demonstrate an ultrasensitive angular interrogation sensor based on the transverse electric (TE) mode surface lattice resonance (SLR) mechanism in an all-metal metasurface. The SLR is from the coupling between the LSPR and the Rayleigh anomaly (RA) diffraction waves^35^ with an ultranarrow resonance linewidth^36^. In sharp contrast to the SPP, the SLR has a prominent character, which can be excited by arbitrary polarization states to form abundant optical phenomena and applications, including multimode SLRs^37^, phase-modulation biosensing^38^, and angular sensing in particular. The TE SLR response will be blueshift (redshift) regularly with the incident angle (RI) increasing, so that we can derive the angular sensitivity function in theory, where we emphasize the influence of the refraction process from the air to the solutions on the resonance wavelength. The experimental and simulated sensitivities can reach 4304.35°/RIU and 6304.35°/RIU respectively, promoting an order of magnitude compared to current angular interrogation SPP-sensors. In the measurement, the broadband light source takes the place of the traditional single-wavelength laser, allowing for flexible wavelength selection and ensuring normal incidence conditions. Moreover, we prove more excellent sensing performance of the designed sensor in the large angle range (0°–90°) than those without considering the refraction and from the TM mode beam. This research provides a novel idea and systematic method for ultrasensitive angular interrogation sensing and exhibits promising prospects in biochemical detection, concentration analysis, etc.

## Results and discussion

### The all-metal metasurface design and theoretical analysis of TE mode SLR

**Fig. 1:**
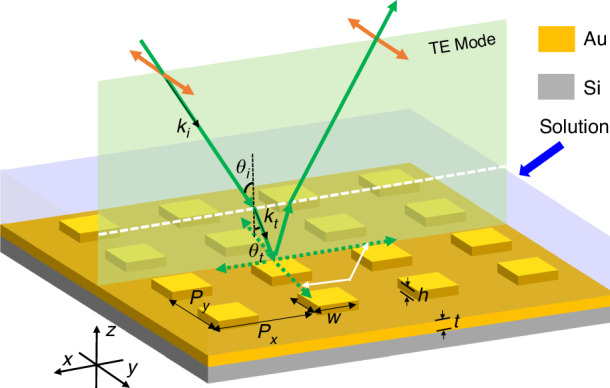
**Schematic diagrams of the proposed angular interrogation sensor based on an all-metal metasurface, where the structural parameters are as follows**: ${P}_{x}$ = ${P}_{y}$ = 700 nm, $w$ = 400 nm, $h$ = 50 nm, $t$ = 120 nm. The x-z plane is defined as the incident plane, and the polarization is parallel to the x-direction

The structural and principal schematic of the proposed ultrasensitive angular interrogation sensor is shown in Fig. 1. The all-metal metasurface comprises a square array of Au meta-atoms atop a thick Au substrate. The lattice length is designed as ${P}_{x}$ = ${P}_{y}$ = 700 nm that plays a significant role in the RA wavevectors and decides the resonance position and wavelength-based sensitivity^39^, and the meta-atom is a square nanorod with $w$ = 400 nm and $h$ = 50 nm influencing the Ohmic losses and SLR linewidths^39^. The substrate is required to be thick enough ($t$ = 120 nm) to avoid transmission losses and support the reflection-type SLR, which can solve the problem of the RI mismatch between the glass substrate and coating liquid layers^40^ compared with the transmission-type SLR. It is worth noting that the Au material has high reflectivity in the near-infrared band and is suitable for biological modification^7^. As for the incident beam, the polarization is parallel to the y-direction and the beam is oblique in the x-z plane which is the incident plane. Since the solution under-tested has a certain liquid level height (about 1 mm scale) rather than being distributed in the full space in the experiment, there is an inevitable refractive process at the air-solution interface, which will reduce the incident angle to improve the sensing property. After being modulated by the metasurface, the reflected beam will refract again at the interface, which will change the outgoing direction but not disturb the SLR spectrum information.

When an incident beam with TE mode propagates obliquely in the x-z plane from air to the water, the beam will generate a refraction phenomenon following Snell’s Law:

1 $${{{k}}}_{{{i}}}\cdot \,\sin {{{\theta }}}_{{{i}}}={{{k}}}_{{{t}}}\cdot \,\sin {{{\theta }}}_{{{t}}}={{n}}\cdot {{{k}}}_{{{i}}}\cdot \,\sin {{{\theta }}}_{{{t}}}$$

where ${k}_{i}$ and ${k}_{t}$ are the incident and refractive wavevectors separately, ${\theta }_{i}$ is the incident angle in the air, ${\theta }_{t}$ is the corresponding transmission angle within the liquid, and $n$ is the RI of the solution. Then, the refractive beam will be modulated by the periodic meta-atoms in the liquid environment to produce RA diffraction waves that propagate parallel to the array plane without z-direction wavevectors^41–43^, and obey the Bragg’s coupling equation^9^:

2 $$|{{{k}}}_{{{RA}}}|=|{-{{k}}}_{{{RA}}}\cdot \,\sin {{{\theta }}}_{{{t}}}\cdot {\boldsymbol{x}}+{{p}}\cdot {{\boldsymbol{R}}}_{{\boldsymbol{x}}}+{{q}}\cdot {{\boldsymbol{R}}}_{{\boldsymbol{y}}}|$$

where the ${k}_{{RA}}$ is the RA wavevector and equivalent to the ${k}_{t}$ in terms of numerical values (${k}_{{RA}}={k}_{t}$), the ${\boldsymbol{x}}$ is the x-axis unit vector, ${{\boldsymbol{R}}}_{{\boldsymbol{x}}}$ and ${{\boldsymbol{R}}}_{{\boldsymbol{y}}}$ are reciprocal lattice vectors of the square lattice in the x- and y-direction ($\left|{{\boldsymbol{R}}}_{{\boldsymbol{x}}}\right|=\left|{{\boldsymbol{R}}}_{{\boldsymbol{y}}}\right|=2\pi /{P}_{y}$), and $p$ and $q$ are the corresponding RA diffraction orders. Generally, we only consider the first-order one that owns a stronger resonance response than others. Because the reflection-type RA is determined by the reciprocal lattice vector parallel to the polarization direction instead of the perpendicular one^39^, the incident TE beam will be regulated by the ${{\boldsymbol{R}}}_{{\boldsymbol{y}}}$, that is $p=0$ and $q=\pm 1$. Hence, we simplify Eq. 2 and derive the RA wavelength ${\lambda }_{{RA}}$ (=$2\pi n/{k}_{{RA}}$) as follows:

3 $${{{\lambda }}}_{{{RA}}}{={{P}}}_{{{y}}}\cdot \sqrt{{{{n}}}^{2}-{\sin }^{2}{{{\theta }}}_{{{i}}}}$$

Obviously, the ${\lambda }_{{RA}}$ will redshift with the increasing $n$, which establishes the theoretical foundation for wavelength-interrogation RI sensing. On the other hand, the ${\lambda }_{{RA}}$ blueshifts in the case of larger ${\theta }_{i},$ which is different from the redshift property of SPP entirely^44^. In addition, the incident beam can also excite the LSPR near the meta-atom, which will couple with the RA waves to generate the SLR response. In essence, the SLR belongs to a special Fano resonance^45^, since the LSPR can be seen as the bright mode and the RA is the dark mode. Due to the LSPR coupling effect, the SLR wavelength ${\lambda }_{{SLR}}$ is a little larger than ${\lambda }_{{RA}}$^33^:

4 $${{{\lambda }}}_{{{SLR}}}={{{\lambda }}}_{{{RA}}}+{\Delta {{\lambda }}_{{LSPR}}={{P}}}_{{{y}}}\cdot \sqrt{{{{n}}}^{2}-{\sin }^{2}{{{\theta }}}_{{{i}}}}+\Delta {{\lambda }}_{{LSPR}}$$

The corresponding redshift increment $\Delta {\lambda }_{{LSPR}}$ is decided by the $w$, $h$, and duty ratio of the meta-atom. Equation 4 exhibits the coupling process between the RA waves and the LSPR, providing an excellent mathematical description approach.

### Experimental and simulated characterization of the TE mode SLR

**Fig. 2:**
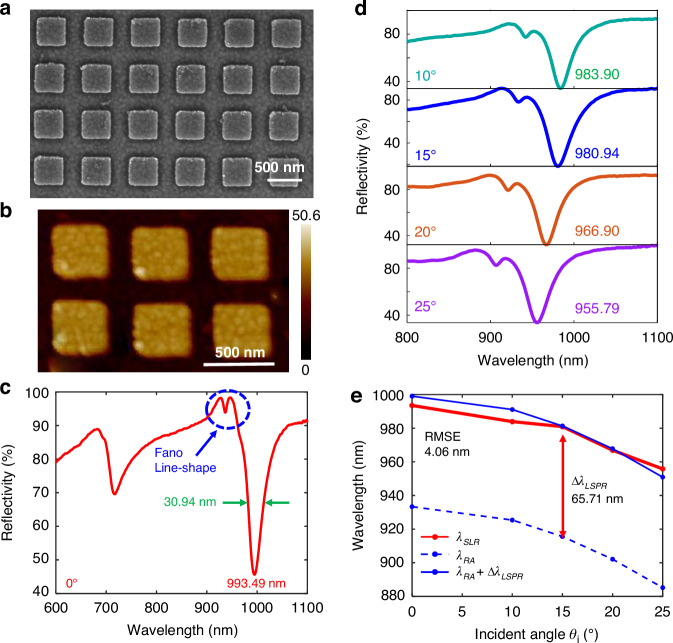
**Experimental results of the TE mode SLR**. **a**, **b** The SEM and AFM images of the fabricated sample. **c** The measured SLR spectrum at ${\theta }_{i}$ = 0°. **d** The SLR spectra from different ${\theta }_{i}$ from 10° to 25° at 5° intervals. **e** The experimental and derived SLR wavelengths, the theoretical RA wavelengths, and the fitting redshift increment $\Delta {\lambda }_{{LSPR}}$. The corresponding simulated results are in Note 1, Supplementary Materials

In the experiment, we fabricated the all-metal metasurface by the sputtering, electron beam lithography, electron beam evaporation, and lifting-off processes. Figures 2a, b are the scanning electron microscope (SEM) and atomic force microscope pictures of the sample separately, exhibiting consistency with the design model. The fabricated metasurface area is 900 × 900 μm^2^, which can contain all parts of the incident light spot with a diameter of 800 μm. We make use of the angular-resolved optical system with a broadband light source (600–1100 nm) to measure the reflectivity in the far field. Considering that water is a popular solvent, hence, the following analysis involves a deionized water ($n$ = 1.3312) layer on the metasurface. In the case of normal incidence, the measured SLR spectrum is shown in Fig. 2c, with a ${\lambda }_{{SLR}}$ of 993.49 nm and a full width at half maximum (FWHM) of 30.94 nm. The asymmetric Fano line-shape proves the couple between the RA (dark mode) and LSPR (bright mode). Although the FWHM can be further narrowed by reduced meta-atom width $w$, the noise resistance and resonance strength of the SLR signal are also weakened, resulting in a lower light-matter interaction. In view of sensing performance, the wider FWHM even improves the angular sensing sensitivity (Detailed simulated results are in Note 3, Supplementary Materials).

With the ${\theta }_{i}$ increasing from 10° to 25°with an interval of 5°, the ${\lambda }_{{SLR}}$ blueshifts from 983.90 nm to 955.79 nm gradually (Fig. 2d). The blueshift trend of the experimental ${\lambda }_{{SLR}}$ (Fig. 2e, red line) is similar to the theoretical RA wavelength (Fig. 2e, blue dashed line) but with a $\Delta {\lambda }_{{LSPR}}$ gap. When the metasurface design remains invariant, the $\Delta {\lambda }_{{LSPR}}$ is also a function of the ${\theta }_{i}$ essentially but without apparent spectrum alteration, so we regard it as a constant in this case for simplification and practicality. The root mean square error (RMSE) value between the measured and derived SLR wavelengths (Fig. 2e, blue solid line) is only 4.06 nm when the $\Delta {\lambda }_{{LSPR}}$ is fitted as 65.71 nm, which agrees well with Eq. 4 strictly. In the case of larger incident angles, the oblique incidence will break the symmetry of the proposed metasurface more seriously, resulting that the LSPR is more sensitivity to the$\,{\theta }_{i}$. Hence, the $\Delta {\lambda }_{{LSPR}}$ will have a larger RMSE, so it is not suitable to consider $\Delta {\lambda }_{{LSPR}}$ as a constant.

**Fig. 3:**
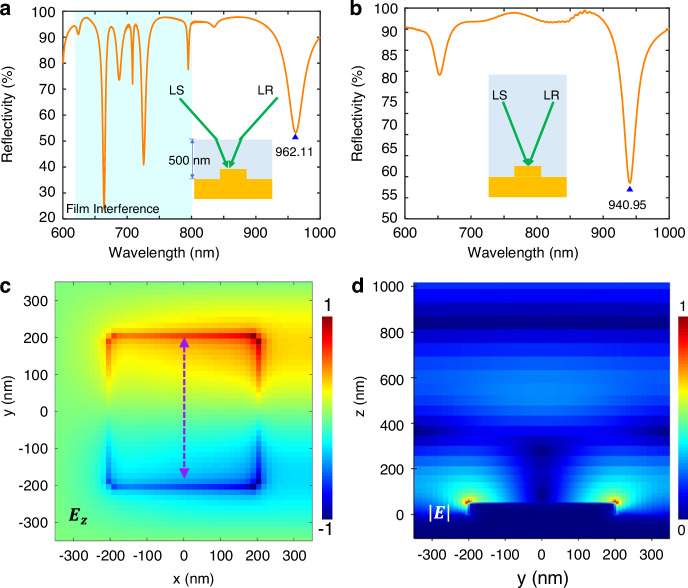
**Numerical simulation results of the TE SLR**. **a** The spectrum of the water medium layer with a finite height (500 nm). The inset is the sketch map involving the refraction. **b** The spectrum when the RI of the full space is 1.3312. The insert schematic only shows reflection phenomenon. **c** The normalized z-direction electric field ${E}_{z}$ distribution at ${\lambda }_{{SLR}}$ = 962.11 nm in the surface of the meta-atom. **d** The normalized total electric field intensity $\left|E\right|$ distribution in the x-z plane

Moreover, we adopt the numerical simulation based on the finite-difference time-domain method to emphasize the influence of the refraction phenomenon on the SLR. When we have added a deionized water layer with a finite height of 500 nm on the top of the metasurface model (Fig. 3a), taking ${\theta }_{i}=$20° as an example, the ${\lambda }_{{SLR}}$ is at 962.11 nm with a relative error of only 0.495% compared with the measured result (966.90 nm). Notably, there are some resonance dips in the 600–800 nm band from the film interference, but they won’t exist in the experiment since the measured liquid heights are milli-scale with a negligible interference effect. In the simulation, the water layer thickness value can disturb the interference response but does not change the SLR wavelength. As for the full medium space simulation without the refractive process, the ${\lambda }_{{SLR}}$ is only 940.95 nm (Fig. 3b), which cannot meet the experimental one precisely. Therefore, the refraction phenomenon in the liquid interface is non-negligible in the subsequent angular interrogation sensing analysis, which will influence the SLR wavelength and the angular sensing performance.

In the case of ${\lambda }_{{SLR}}$ = 962.11 nm, opposite charges from z-direction electric field ${E}_{z}$ are distributed at the edge of the meta-atom along the y direction (Fig. 3c), which exbibits the periodicity and directionality of traveling waves and means the RA waves propagate parallel to the polarization^40^. The corresponding SLR mode is modulated by the ${P}_{y}$ rather than ${P}_{x}$, belonging to (0, ±1) orders (Eq. 4). By the way, the asymmetric ${E}_{z}$ distribution in the x direction results from the oblique incidence. Due to the TE mode polarization state, the RA wavevector propagates perpendicular to the incident wavevector, leading to a prominent difference compared to the SPP. The total electric field intensity $\left|E\right|$ localizes on the edge of the meta-atom mainly in the same direction as the TE mode (Fig. 3d).

### Angular interrogation sensing theory and measurement

According to Eq. 4, the ${\lambda }_{{SLR}}$ will be redshift with the RI of the solution increasing, so that the incident TE beam needs to illuminate the sample with larger ${\theta }_{i}$ in the angular interrogation sensing process to maintain the SLR wavelength ${\lambda }_{{SLR}}$ at the working single wavelength ${\lambda }_{0}$ still (${\lambda }_{{SLR}}={\lambda }_{0}$) and define the SLR angle ${\theta }_{{SLR}}$. Where the ${\theta }_{{SLR}}$ is a special ${\theta }_{i}$ that can be recognized by finding the minimal reflectivity position in the spectrum, because the ${\theta }_{{SLR}}$ can meet the wavevector match to support the SLR and localize the reflection energy. The angular sensitivity ${S}_{\theta }$ is defined as the ${\theta }_{{SLR}}$ increment upon a unit change in the RI to evaluate sensing performance^46^. In this case, the ${S}_{\theta }$ can be derived by the implicit differentiation method for Eq. 4, where both ${\lambda }_{{RA}}$ (=${\lambda }_{{SLR}}$-$\Delta {\lambda }_{{LSPR}}$ = ${\lambda }_{0}$-$\Delta {\lambda }_{{LSPR}}$) and ${P}_{y}$ are constants:

5 $${{\rm{S}}}_{{{\theta }}}=\left|\frac{{{{d}}{{\theta }}}_{{{SLR}}}}{{{dn}}}\right|=\frac{2{{n}}}{\sin (2{{{\theta }}}_{{{SLR}}})}{\rm{rad}}/{\rm{RIU}}=\frac{2{{n}}}{\sin (2{{{\theta }}}_{{{SLR}}})}\cdot \frac{{180}^{\circ}} {\pi }/{\rm{RIU}}$$

As we can see, the ${S}_{\theta }$ is influenced by the $n$ and ${\theta }_{{SLR}}$ without any other factor in principle under the condition that the metasurface structure remains unchanged. The value of $n$ is about 1–2 commonly, which won’t improve the ${S}_{\theta }$ significantly. By contrary, the ${S}_{\theta }$ shall approach infinite at ${\theta }_{i}=$ 0° or ${\theta }_{i}=$ 90°, so that the ${\theta }_{{SLR}}$ plays an extremely important role in the angular sensing. Equation 5 provides a theoretical foundation for the ultrahigh sensitivity feature in the proposed angular sensor technology.

**Fig. 4:**
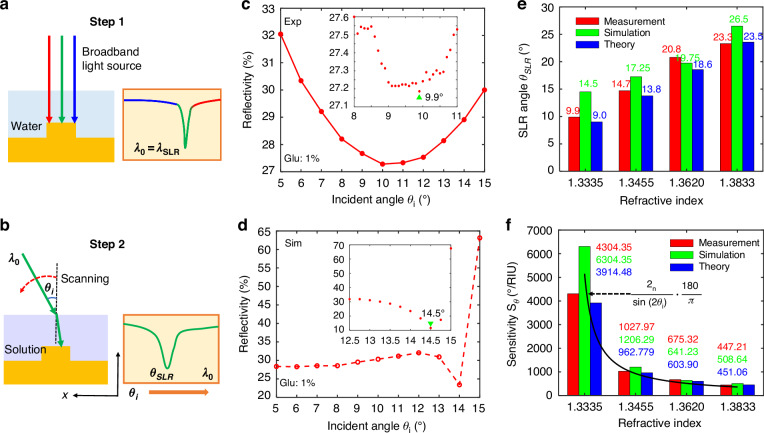
**Angular interrogation sensing results**. **a**, **b** The angular interrogation sensing steps. Step 1: Define the ${\lambda }_{{SLR}}$ at ${\theta }_{i}=$0° as the measurement wavelength ${\lambda }_{0}$; Step 2: Scan the ${\lambda }_{0}$ to catch the ${\theta }_{{SLR}}$. **c**, **d** Experimental and simulated reflectivity spectra of the 1% glucose solution at different ${\theta }_{{SLR}}$. The difference between both reflectivity trends is from the different ${\theta }_{{SLR}}$ and the influence of measured and fabricated errors. But both reflectivity values reduce first and increase later near the ${\theta }_{{SLR}}$. **e**, **f** The SLR angles and angular sensitivities of different solutions from measurement, simulation, and theory derivation. Detail reflectivity-${\theta }_{{SLR}}$ plots can be found in Note 4, Supplementary Materials

In general, normal incidence is the most common and simplest measurement condition. However, the traditional single-wavelength laser cannot produce the SLR exactly under normal incidence due to fabrication errors. For example, the simulated TE SLR wavelength is 984.03 nm at ${\theta }_{i}=$ 0°, but the measured one is 993.49 nm, which means that we cannot fabricate the ideal sample to meet the design and match the laser wavelength perfectly. So the oblique incidence is required to blueshift the ${\lambda }_{{SLR}}$ to make the ${SLR}$ happened in the laser wavelength as depicted in Eq. 4. Unfortunately, a larger ${\theta }_{{SLR}}$ will reduce the angular sensitivity based on Eq. 5. To solve the problem, we adopt a broadband light source in the experiment to take place the conventional laser wavelength. We can define the measured ${\lambda }_{{SLR}}$ in the case of ${\theta }_{i}=$ 0° as the working single wavelength ${\lambda }_{0}$ directly (Fig. 4a), which breaks the limitations of laser sources and decreases the design and fabrication requirement. Besides, this improvement can be applied to all kinds of metasurface designs and is always able to meet the normal incidence condition to realize ultrasensitive angular sensing, with more flexibility and compatibility. In the following measurement, therefore, we only focus on the wavelength of 993.49 nm based on the SLR results in Fig. 2c. The second step is scanning the ${\theta }_{i}$ in the different solutions to gain the corresponding ${\theta }_{{SLR}}$ (Fig. 4b).

We take glucose solutions with different concentrations as examples to analyze the proposed angular interrogation sensing performance. The glucose solutions at 1%, 10%, 20%, and 30% in weight ratio are dropped on the sample surface, and the corresponding RI values are 1.3335, 1.3455, 1.3620, and 1.3833, respectively. As shown in Fig. 4c, we preliminarily estimate the ${\theta }_{{SLR}}$ range of the 1 wt% solution from 8° to 11° by varying the incident direction at 1° interval, and then scan the range precisely with an interval of 0.1° to obtain an accurate ${\theta }_{{SLR}}$ of 9.9°. Similarly, the simulated ${\lambda }_{{SLR}}$ (${\lambda }_{0}$) is 984.03 nm, leading to an angle change of 14.5° larger than that of measurement owing to ideal and lossless models (Fig. 4d). In addition, the theoretical ${\theta }_{{SLR}}$ calculated based on Eq. 4 is 9.0°, agreeing well with the measurement. The measured, simulated, and theoretical SLR angles of other concentration solutions are shown in Fig. 4e. Three kinds of ${\theta }_{{SLR}}$ results are matched with each other precisely and all of them will become larger with the RI increasing. For example, the experimental ${\theta }_{{SLR}}$ changes from 9.9° to 23.3° gradually.

In Fig. 4f, the measured ${S}_{\theta }$ can reach 4304.35°/RIU at most, improved by about an order of magnitude compared to the current results of other angular interrogation sensors (see Note 2, Supplementary Materials for details). The corresponding RI resolution is 2.32$\times$10^-5^ RIU, better than the performance of the Abbe refractometer and comparable to that of the SPP sensor. Of course, we can further optimize the resolution based on a smaller angle interval. The simulated ${S}_{\theta }$ for the 1 wt% solution is up to 6304.35°/RIU, providing numerical support for the ultrasensitivity sensing. Theoretically, Eq. 5 can explain this ultrahigh sensitivity phenomenon that originates from the ${\theta }_{{SLR}}$ approaching 0°. Moreover, Eq. 5 can also describe the decreasing trend of ${S}_{\theta }$ values of measurement and simulation accurately from 4304.35 and 6304.35 to 448.08 and 509.62, respectively, resulting from the RI enhancement caused larger ${\theta }_{{SLR}}$. In order to promote the angular sensitivity of high RI solutions, an effective approach is to utilize another higher RI reagent to take the place of water as the reference. Nevertheless, Eq. 5 is a differential expression so it is not suitable for the discrete RI condition in the experiment. The theoretical results in Fig. 4f are adopted the difference method based on the ${S}_{\theta }$ definition, matched greatly with the measurement. For instance, the calculated ${S}_{\theta }$ value at $n=1.3335$ is 3914.48°/RIU with a relative error of only 9.05%.

Another, we analyze the angular sensitivity features influenced by the lattice period and meta-atom width. The numerical simulation results reveal that the ${S}_{\theta }$ is about to reduce a little with the ${P}_{y}$ enlarging or $w$ abating, since the smaller duty ratio leads to a diminished LSPR increment. The details of the result analysis are described in Note 3, Supplementary Materials.

### Sensitivity property analysis

**Fig. 5:**
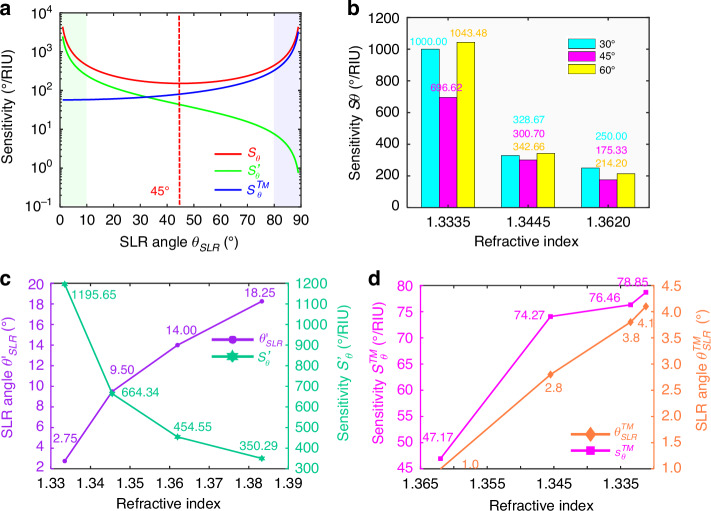
**Three kinds of sensitivity characteristic analysis in the case of wide-angle incidence**. **a** Three sensitivity functions (${S}_{\theta }$, ${S}_{\theta }^{{\prime} }$, and ${S}_{\theta }^{{TM}}$) in the 0°–90° range. The green and blue areas represent the ${\theta }_{{SLR}}$ near the ${0}^{^\circ }$ and ${90}^{^\circ }$, respectively. **b** The experimental ${S}_{\theta }$ results at different incident angles (30°, 45°, and 60°) and different RI solutions (1.3335, 1.3455, and 1.3620). **c** The simulated SLR angle ${\theta }_{{SLR}}^{{\prime} }$ and sensitivity ${S}_{\theta }^{{\prime} }$ without considering the refraction effect. **d** The measured SLR angle ${\theta }_{{SLR}}^{{TM}}$ and sensitivity ${S}_{\theta }^{{TM}}$ of the TM mode SLR sensing

After we analyze the angular sensing characteristics of normal incidence, we move on to explore sensing regularity for the wide-angle incidence. The ${S}_{\theta }$ is a symmetric function about the line of ${\theta }_{{SLR}}=$ 45°, and it will decrease in the range of 0°–45° and increase with the ${\theta }_{{SLR}}$ changing from 45° to 90° (Fig. 5a, red line). According to Eq. 5, when the ${\theta }_{{SLR}}$ is close to 0° or 90°, the ${S}_{\theta }$ can approach infinite as shown in the green and blue areas in Fig. 5a. Hence, the maximal ${S}_{\theta }$ value is not only at 0° but also 90°, and the minimal one is at 45° theoretically. Figure 5b shows the experimental ${S}_{\theta }$ results under the ${\theta }_{i}$ of 30°, 45°, and 60°, where we first measure the ${\lambda }_{0}$ of the above three ${\theta }_{i}$ at the water environment, and then scan the ${\theta }_{i}$ to obtain the ${\theta }_{{SLR}}$. As a whole, the low-RI solutions also have more perfect sensitivities than high-RI ones, but all ${S}_{\theta }$ values are smaller than those of ${\theta }_{i}=$ 0°. The ${S}_{\theta }$ at the ${\theta }_{i}$ of 30° and 60° are nearly consistent under three RI environments, meeting the above symmetry demonstration. In contrast, the sensing property at the ${\theta }_{i}$ of 45° is the most terrible, and the sensitivity is just 175.33°/RIU at $n=1.362$0. In a word, it is the best choice to achieve ultrasensitive angular sensing under normal incidence, which demonstrates the significance of Step 1 in Fig. 4a.

If we don’t consider the refraction effect (${k}_{i}$ = ${k}_{t}$, ${\theta }_{i}$ = ${\theta }_{t}$), Eq. 2 can be simplified as Eq. 6 to express the RA wavelength ${\lambda }_{{RA}}^{{\prime} }$, and the corresponding angular interrogation sensitivity ${S}_{\theta }^{{\prime} }$ can be derived (Note 5, Supplementary Materials) as Eq. 7^47^:

6 $${{{\lambda }}}_{{{RA}}}^{{{{\prime} }}}={{n}}{\cdot} {{{P}}}_{{{y}}}{\cdot} \,{\cos} {{{\theta }}}_{{{i}}}$$

7 $${S}_{\theta }^{{\prime} }=\frac{1}{n\cdot \tan {\theta }_{{SLR}}}\cdot \frac{{180}^{\circ }}{\pi }/{\rm{RIU}}$$

The ${S}_{\theta }^{{\prime} }$ is a subtraction function from 0° to 90° (Fig. 5a, green line), and the part in the 45°–90° range is totally different from the ${S}_{\theta }$. Also, the ${S}_{\theta }^{{\prime} }$ is always smaller than ${S}_{\theta }$ in the same RI environment (Fig. 5c). Taking *n* = 1.3620 as an example, the simulated ${\theta }_{{SLR}}^{{\prime} }$ in full space RI environment is only 14.0°, resulting in a ${S}_{\theta }^{{\prime} }$ of 454.54°/RIU which is 29.1% lower than ${S}_{\theta }$ (641.23°/RIU). Notably, the ${S}_{\theta }^{{\prime} }$ is also close to infinity at ${\theta }_{{SLR}}^{{\prime} }=$ 0° but still cannot reach the identical level, demonstrated by Eq. 8^48^:

8 $$\mathop{{\lim}}\limits_{{{\theta}}_{{SLR}}\to {{0}^{\!{\circ}\, }}}\frac{{{\rm{S}}}_{{{\theta }}}}{{{\rm{S}}}_{{{\theta }}}^{{{{\prime} }}}}=\mathop{{\lim}}\limits_{{{{\theta }}}_{{{SLR}}}\to {{0}^{\!{\circ}}}}\,\frac{2{{n}}}{\sin (2{{{\theta }}}_{{{SLR}}})}{\cdot} {{n}}{\cdot} \,\tan {{{\theta }}}_{{{i}}}={{{n}}}^{2}\,{{>}}\,1$$

As a result, the refraction process can not only keep the consistency between the measurement and the theory but also improve the sensing effect with advantage.

What’s more, the incident beam with TM mode polarization (RA diffraction order: *p* = +1, *q* = 0) can also realize angular interrogation sensing, whose RA wavelength ${\lambda }_{{RA}}^{{TM}}$ and sensitivity ${S}_{\theta }^{{TM}}$ are calculated as follow^47^:

9 $${{{\lambda }}}_{{{RA}}}^{{{TM}}}={{{P}}}_{{{x}}}{\cdot} ({{n}}+\,\sin {{{\theta }}}_{{{i}}})$$

10 $${{{S}}}_{{{\theta }}}^{{{TM}}}=\frac{1}{\cos {{{\theta }}}_{{{SLR}}}}\cdot \frac{{180}^{\circ}}{\pi} /RIU$$

where both equations involve the refracion process (See Note 6, Supplementary Materials for details). The ${S}_{\theta }^{{TM}}$ is a monotone-increasing function of the ${\theta }_{{SLR}}$ (Fig. 5a, blue line), and has similar property of the SPP^28^. The minimal ${S}_{\theta }^{{TM}}$ is at ${\theta }_{{SLR}}=$ 0°, opposite to the ${S}_{\theta }$ completely. Since the TM SLR wavelength ${\lambda }_{{SLR}}^{{TM}}$ will be redshift with the ${\theta }_{i}$ rising instead of blueshift based on Eq. 9 (Note 7, Supplementary Materials), the SLR phenomenon won’t appear at any beam angle in glucose solutions in the measurement if we still define the ${\lambda }_{{SLR}}^{{TM}}$ from normal incidence in the water environment as the incident wavelength ${\lambda }_{0}$ (because the ${\lambda }_{0}$ is smaller than required ${\lambda }_{{SLR}}^{{TM}}$ all the time). In this case, we choose to measure the ${\lambda }_{{SLR}}^{{TM}}$ in the 30 wt% glucose solution environment (*n* = 1.3833), which is 1024.37 nm as the ${\lambda }_{0}$. Although both of the ${\theta }_{{SLR}}$ and the ${S}_{\theta }^{{TM}}$ are larger with the RI decreasing (Fig. 5d), the ${S}_{\theta }^{{TM}}$ values are more than one order of magnitude smaller than ${S}_{\theta }$. On the other hand, the ${S}_{\theta }^{{TM}}$ can be optimized by enlarging the oblique incident angle, however, which will make measurement difficult and lack the practicality. It is worth noting that the ${S}_{\theta }^{{TM}}$ is still lower than ${S}_{\theta }$ even if at ${\theta }_{{SLR}}=$ 90° in principle^48^:

11 $$\mathop{{\lim}}\limits_{{{{\theta }}}_{{{SLR}}}\to {{90}^{\!{{\circ}} }}}\,\frac{{{{S}}}_{{{\theta }}}}{{{{S}}}_{{{\theta }}}^{{{TM}}}}=\mathop{{\lim}}\limits_{{{{\theta }}}_{{{SLR}}}\to {{90}^{\!{\circ}}}}\,\frac{2{{n}}}{\sin (2{{{\theta }}}_{{{SLR}}})}{\cdot} \,{\cos}\, {{{\theta }}}_{{{i}}}={{n}}\,{\boldsymbol{ > }}\,1$$

Thereby, the angular interrogation sensing based on the TE mode has a better performance than that of the TM mode, exhibiting the importance of polarization mode for the SLR sensing performance.

## Conclusion

In conclusion, we have demonstrated an ultrasensitive angular interrogation sensing method based on the reflection-type SLR with TE mode polarization in an all-metal metasurface. The TE mode SLR wavelength will be blueshift regularly with the incident angle increasing as the description of Eq. 4. And the derived angular sensitivity involves the refraction process at the air-solution interface, which is a non-negligible factor and can influence the SLR position in the spectrum and further optimize the sensitivity. The sensitivity is determined by the SLR angle mainly and can reach infinity in theory at normal incidence, providing theoretical support for the ultra-high sensing performance. In the experiment, we utilize broadband light instead of a laser as the excitation source and define the measured SLR wavelength at ${\theta }_{i}=$ 0°as the working single wavelength, breaking the limitations of single wavelength from laser light sources, ensuring the normal incidence condition at all times, and thus reducing design and fabrication difficulties of the metasurface. The highest measured sensitivity reaches 4304.35°/RIU, promoting an order of magnitude compared with current results based on the TM mode SPP mechanism. The measured sensitivity will be smaller in part with the RI or incident angle increasing owing to a larger normal direction deviation but still more excellent than traditional ones. In the wide-angle case, the performance of the proposed sensor is better than that of other sensors which are without analyzing the refraction or excited by the TM-polarized beam. Actually, this research not only constructs a new theory of ultrasensitive angular interrogation sensing but also proposes a SLR-metasurface device platform, which will undoubtedly open up a monumental path for biochemical detection in solution environments.

## Materials and methods

### Metasurface fabrication

The proposed all-metal metasurface has a simple geometric structure and does not require complex processes. First of all, a 120-nm-thick layer of Au was deposited by magnetron sputtering on a clean silicon substrate. The Au layer was then treated with reactive oxygen plasma (60 W, 0.3 Torr, 10 s) to improve its surface hydrophilicity. Secondly, the electron-beam (e-beam) resist (PMMA AR-P 679.04) was spin-coated on the Au surface at 4000 rpm for 60 s and baked in the hot plate at 170 °C for 3 min. In the next steps to fabricate meta-atoms, the e-beam lithography with the 8567.2 pA current and $500\,{\rm{\mu}} {\rm{C}}/{{\rm{cm}}}^{2}$ doses was employed to define the square nanorod with a width of 400 nm. The exposed patterns were developed in the developer (AR 600-55) for 1 min and were later washed with the fixing solution (Isopropanol) for 0.5 min. Subsequently, an Au layer with a 50-nm-thickness was deposited by e-beam evaporation with a vacuum degree of about 10^-8 ^Torr and a deposited speed of 0.5 Å/s. Finally, the redundant Au material on the PMMA was lifted off in acetone solution for 4 h, followed by cleaning and drying.

### Spectrum measurement

We measured various SLR spectra at different angles by a macro angular-resolved spectroscopy testing system, which is composed of a halogen light source (400–1100 nm), a spectrometer (Wavelength resolution: 0.76 nm), a polarizer (Extinction ratio: 100:1), a light path (That includes the incident port and receive port. The diameter of the light spot is about 800 μm.), and an angle-rotation control system (Angle resolution: 0.1°, angle range: 0°–180°).

### Finite-difference time-domain simulations

We designed the proposed metasurface model firstly, including the Au substrate, Au meta-atom, and the under-tested solution with a 500-nm height, where we only considered the RI feature of the solution without involving absorb losses. The permittivity of Au is taken from Palik. A mesh grid with a maximum cell size of 2 nm was defined near the meta-atom for high simulation accuracy. The structures were illuminated by a plane wave light source with a broad waveband, whose incident angle could be changed flexibly. In particular, the source was required to adopt broadband fixed-angle source technology in the case of an oblique incident of broadband light. The simulation unit adopted periodic boundary conditions at x- and y-directions and the perfectly matched layers were employed along the z-direction. On the other hand, when we sweep the SLR wavelength at different incident angles, the x- and y-direction boundary conditions should be tuned as Bloch conditions. Finally, to analyze various simulation results in detail, we should add monitors in the near- and far-field.

## Supplementary information

Supplement Material
