# Supplementary material for: An ultrasensitive angular interrogation metasurface sensor based on the TE mode surface lattice resonance
Source: Microsyst Nanoeng. 2025 Jan 8;11:1. doi: 10.1038/s41378-024-00848-5 (PMC11707350; doi:10.1038/s41378-024-00848-5)
Supplement: Supplementary file 1 — Supplement Material [file 41378_2024_848_MOESM1_ESM.docx]

Supplementary Materials

An ultrasensitive angular interrogation metasurface sensor based on the TE mode surface lattice resonance

*Liye Li^1,2^, and Wengang Wu^1,2,3,4*^*

1. National Key Laboratory of Advanced Micro and Nano Manufacture Technology, Beijing 100871, P. R. China
2. School of Integrated Circuits, Peking University, Peking University, Beijing 100871, P. R. China
3. Beijing Advanced Innovation Center for Integrated Circuits, Beijing 100871, P. R. China
4. Frontiers Science Center for Nano-optoelectronics, Peking University, Beijing 100871, P. R. China

*Corresponding authors: [wuwg@pku.edu.cn](mailto:wuwg@pku.edu.cn);

**Note 1: Simulated spectra of TE beams with different incident angles**

We have adopted the numerical simulation based on the finite-difference-time-domine method to support the experiment results shown in Figure 2. As the incident angle $\theta_{i}$ of the transverse electric (TE) beam increases from 0° to 25°, the simulated surface lattice resonance (SLR) wavelength $\lambda_{SLR}$ will be also blueshift from 984.03 nm to 952.75 nm generally (Figure S1a), with identical trend as the experimental values (Figure S1b). The root mean square error (RMSE) is only 7.79 nm between simulated and measured results, resulting from the fabrication errors and the disturbance of film interference in the simulation (Figure S1b, orange part). Similarly, the simulated $\lambda_{SLR}$ is still larger than the theoretical Rayleigh anomaly (RA) wavelength $\lambda_{RA}$ from the coupling with the localized surface plasmon resonance (LSPR). When the LSPR increment ${\Delta\lambda}_{LSPR}$ is defined as 56.89 nm, the derived SLR wavelength ($\lambda_{RA}+{\Delta\lambda}_{LSPR}$) can fit the simulated values precisely, with a small RMSE of 6.03 nm.

**
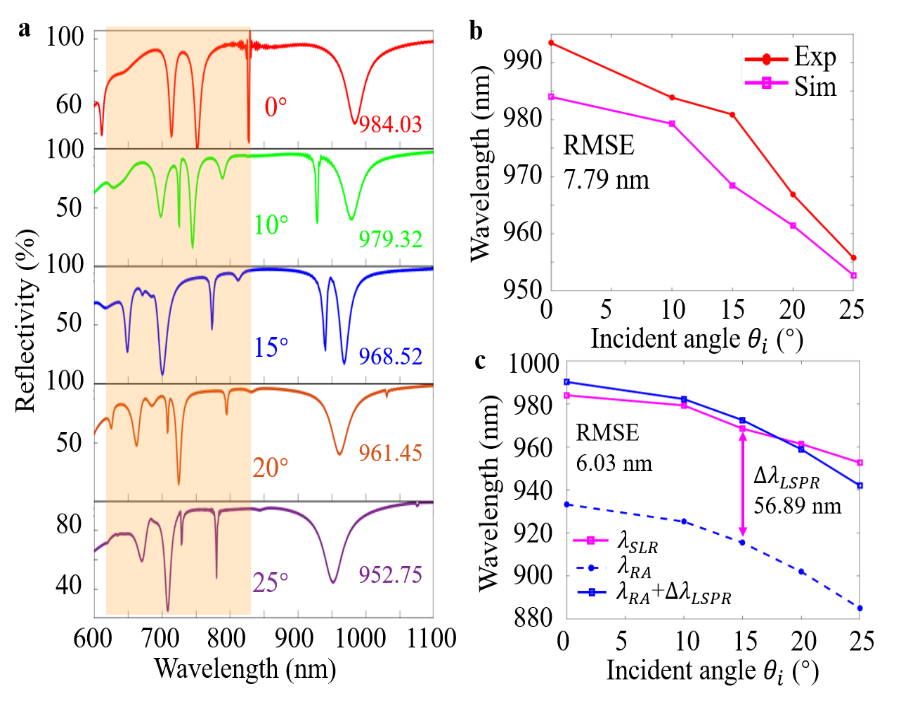
**

**Figure S1.** Simulated results of the TE SLR. **a.** The simulated spectra of TE beams with different $\theta_{i}$ from $0^{^{\circ}}$ to ${25}^{^{\circ}}$. **b.** The comparison between the simulated and the measured wavelength $\lambda_{SLR}$. **c,** The theoretical RA wavelength $\lambda_{RA}$, the simulated $\lambda_{SLR}$, the derived SLR wavelength ($\lambda_{RA}+{\Delta\lambda}_{LSPR}$), and the fitting LSPR increment $\Delta\lambda_{LSPR}$.

**Note 2:** **Sensitivity comparison of current angular interrogation sensors**

At present, the vast majority of angular interrogation sensors are based on the surface plasmon polariton (SPP) mechanism, which is from the prism- or grating-coupling. Although numerous research studies have proposed various SPP devices to improve the sensing sensitivity, there has been no significant effect. The main reason is that the SPP property has already determined the sensing performance substantially. For example, the incident beam is always required to have a large oblique angle to excite SPP and the angular sensitivity values are thus lower than 1000°/RIU all the time, as shown in Table S1. In this work, we have adopted a new theory to achieve angular sensing, with essential creativity. The SLR in an all-metal Au-based metasurface excited by the TE-polarized broadband beam does not require the large-angle incidence, and the measured sensitivity can reach 4304.35°/RIU with an order of magnitude improvement.

**Table S1.** Sensitivity comparison of the proposed sensor with other SPP-based sensors

| Reference | Structure | Mechanism | Incident angle (°) | Sensitivity (°/RIU) |
| --- | --- | --- | --- | --- |
| Ashrafi et al. (2022)^1^ | prism/Ag/GaN/WS_2_ | SPP | 55 - 70 | 186.59 |
| Hu et al. (2011)^2^ | Au/Al grating | SPP | 55 - 70 | 187 |
| Kong et al. (2020)^3^ | prism/Ag grating /graphene | SPP | 70 - 80 | 220.67 |
| Cai et al. (2008)^4^ | Au grating | SPP | 60 - 70 | 237 |
| Mishra et al. (2016)^5^ | prism/Rh/Ag  /Si/graphene | SPP | 70 - 80 | 240 |
| Su et al. (2012)^6^ | Al grating | SPP | 65 - 75 | 247.2 |
| Bijalwan et al. (2017)^7^ | Ag/Au grating | SPP | 75 - 85 | 346 |
| Nur et al. (2019)^8^ | prism /Ag  /BP/WSe_2_ | SPP | 75 - 85 | 367 |
| Wang et al. (2023)^9^ | Au/Ag grating | SPP | 65 - 85 | 443.5 |
| Vibisha et al. (2023)^10^ | prism/Cu/BP | SPP | 75 - 87 | 628 |
| Wang et al. (2018)^11^ | prism/Au | SPP | 70.2 - 76.2 | 735 |
| This work | **All-metal Au-based metasurface** | **TE mode SLR** | **0 - 23.3** | **4304.35** |

**Note 3:** **The influence of the metasurface design on the angular sensitivity**

As shown in Figure S2a, taking $n=1.3620$ as an example, the SLR angle $\theta_{SLR}$ will reduce from 19.75° to 16.25° when the period length $P_{y}$ of the meatsurface enlarges from 700 nm to 900 nm at an interval of 100 nm and, consequently, the angular sensitivities $S_{\theta}$ are 641.23°/RIU, 568.18°/RIU, and 527.59°/RIU, respectively. On the other hand, the decreasing meta-atom width $w$ will also diminish the $\theta_{SLR}$ and $S_{\theta}$, as described in Figure S2b, where the minimal $\theta_{SLR}$ and $S_{\theta}$ are 17.5°, and 568.18°/RIU.

In theory, above two kinds phenomena are from the reduction of the LSPR increment $\Delta\lambda_{LSPR}$, as follows:

$\left(\frac{\text{λ}_{\text{SLR}}\text{-}\Delta\text{λ}_{\text{LSPR}}}{\text{P}_{\text{y}}}\right)^{2}\text{=n}^{2}\text{-}\sin^{2}\text{θ}_{\text{SLR}}$ (S1)

Equation S1 is another express of Equation 3, where $\lambda_{SLR}$ and $n$ are constants. Both of a larger $P_{y}$ and a smaller $w$ will abate the duty cycle of the meta-atom, with less Ohmic losses. Hence, the $\Delta\lambda_{LSPR}$ is smaller and the $\theta_{SLR}$ will be also diminished accordingly based on Equation S1. Then, the small $\theta_{SLR}$ leads to a decrescent $S_{\theta}$.

**
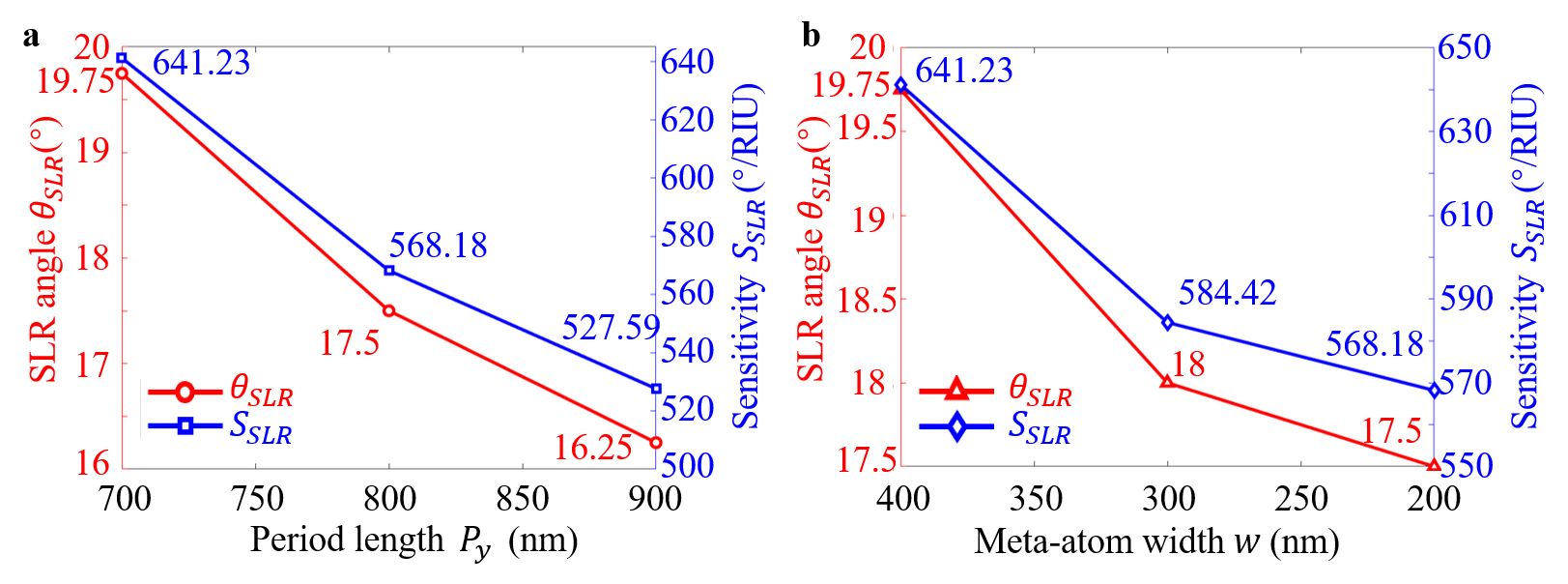
**

**Figure S2.** The influence of the metasurface design on the SLR angle and the angular sensitivity. The $\theta_{SLR}$ and $S_{\theta}$ changes with **a.** the period length $P_{y}$ increasing and **b.** the meta-atom width $w$ decreasing of the matesurface.

**Note 4: Measured and simulated SLR angles of other glucose solutions**

In the measurement, we first scan the SLR wavelength of 993.49 nm in a wide-angle range with an interval of 1° to detect the SLR angle approximately, and then scan more finely with 0.1° intervals. The SLR angles of glucose solutions with 10, 20, and 30 wt% are 14.7°, 20.8°, and 23.3° (Figure S3a, c, e), respectively. Besides, the corresponding simulated SLR angles of the three kinds of solutions are 17.25°, 19.75°, and 26.5° based on the incident-angle scanning under the wavelength of 984.03 nm (Figure S3b, d, f). The angle intervals in the numerical simulation are 1° and 0.25°.

**
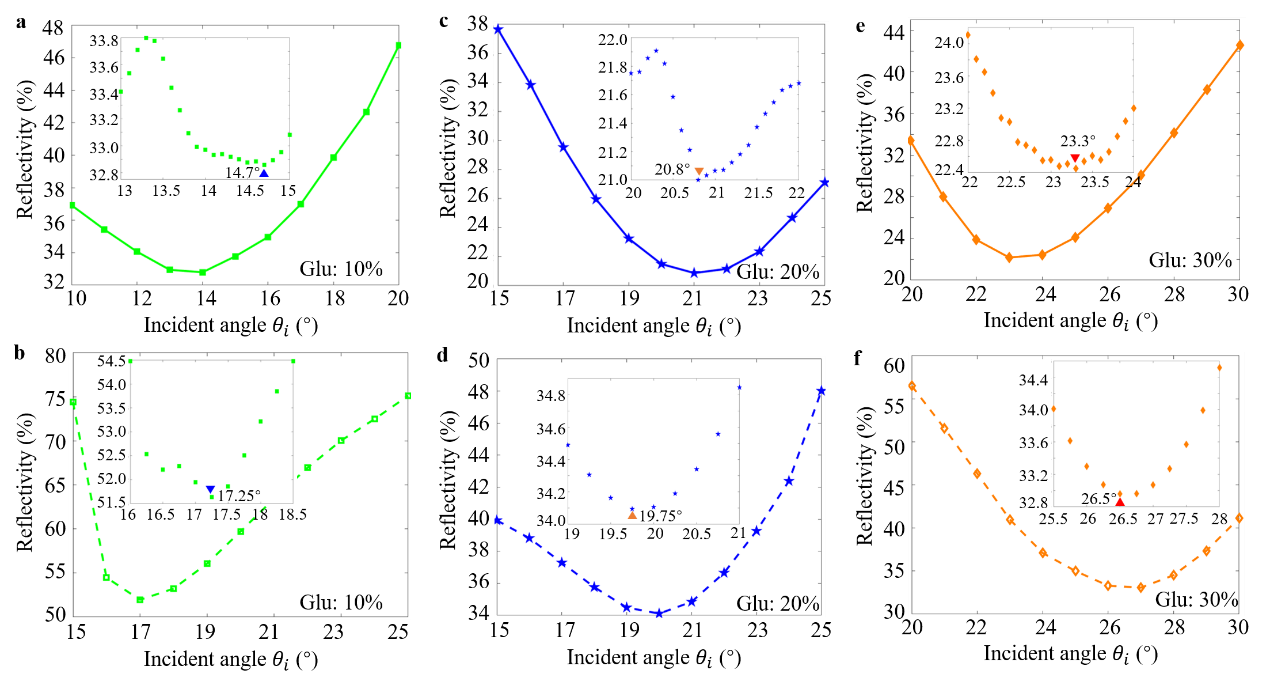
**

**Figure S3.** Measured and simulated SLR angles of three kinds of glucose solutions with different concentrations. **a. c. e.** The measured plots between the incident angle $\theta_{i}$ and the reflectivity of glucose solutions with 10, 20, and 30 wt% respectively. The angle intervals in the experiment are 1° and 0.1° separately. **b. d. f.** The corresponding simulation results of above glucose solutions. The angle intervals in the numerical simulation are 1° and 0.25°.

**Note 5: Derivation of the sensitivity function in the full refractive index space**

In the full refractive index (RI) space, there is not refraction process when the incident beam illuminates the metasurface sample, so that Equation 2 can be changed as:

$\left|\text{n}\cdot\text{k}_{\text{i}}\right|\text{=}\left|\text{-n}\cdot\text{k}_{\text{i}}\cdot\sin\text{θ}_{\text{i}}\cdot\overrightarrow{\text{x}}\text{+p}\cdot R_{x}\text{+q}\cdot R_{y}\right|$ (S2)

where the $k_{i}$ is the incident wavevector in the air, $n$ is the RI of the full space, the $\theta_{i}$ incident angle in the full RI space, and the other parameters are same as the Equation 1. Next, Equation S2 can be simplified due to $p=0$ and $q=\pm1$, as follows:

$\left|\text{n}\cdot\text{k}_{\text{i}}\right|\text{=}\left|\text{-n}\cdot\text{k}_{\text{i}}\cdot\sin\text{θ}_{\text{i}}\cdot\overrightarrow{\text{x}}\pm R_{y}\right|=\sqrt{\left(\text{-n}\cdot\text{k}_{\text{i}}\cdot\sin\text{θ}_{\text{i}}\right)^{2}+\left(\pm2\pi/\text{P}_{y}\right)^{2}}$ (S3)

Since the RA wavelength $\lambda_{RA}^{'}$ is calculated as ${2\pi}/{k_{i}}$, which can be substituted into Equation S3:

$\left(\text{n}\cdot\frac{2\pi}{\lambda_{\text{RA}}^{'}}\right)^{2}\text{=}\left(\text{-n}\cdot\frac{2\pi}{\lambda_{\text{RA}}^{'}}\cdot\sin\text{θ}_{\text{i}}\right)^{2}+\left(\frac{2\pi}{\text{P}_{\text{y}}}\right)^{2}$ (S4)

Finally, we can obtain the $\lambda_{RA}^{'}$ function (That is Equation 5) by some simplification steps:

$\lambda_{\text{RA}}^{'}=\text{P}_{\text{y}}\cdot\sqrt{\text{n}^{2}-\text{n}^{2}\cdot\sin^{2}\text{θ}_{\text{i}}}=\text{n}\cdot\text{P}_{\text{y}}\cdot\cos\text{θ}_{\text{i}}$ (S5)

As for the angular sensitivity $S_{\theta}^{'}$ is derived by the implicit differentiation from Equation S5, where the $\lambda_{RA}^{'}$ and $P_{y}$ are constants, and the $\theta_{i}$ is replaced by the SLR angle $\theta_{SLR}$:

$0=\frac{d\left(\text{n}\cdot\text{P}_{\text{y}}\cdot\cos\text{θ}_{\text{SLR}}\right)}{d\text{n}}=\text{P}_{\text{y}}\cdot\left(\cos\text{θ}_{\text{SLR}}\text{-n}\cdot\sin\text{θ}_{\text{SLR}}\cdot\frac{d\text{θ}_{\text{SLR}}}{d\text{n}}\right)$ (S6)

Furthermore, the angular sensitivity $S_{\theta}^{'}$ function requires a unit change from radians to angles as follows:

 (S7)

**Note 6: Derivation of the sensitivity function of the TM SLR**

The polarization of transverse magnetic (TM) mode decides that the RA waves will propagate along the x-direction, so the SLR is influenced by the $P_{x}$, resulting in $p=\pm1$ and $q=0$. Hence, Equation 2 is described as a new express:

$\left|\text{k}_{\text{t}}\right|\text{=}\left|\text{-k}_{\text{t}}\cdot\sin\text{θ}_{\text{t}}\cdot\overrightarrow{\text{x}}\text{ }\pm R_{x}\right|$ (S8)

where $k_{t}=n\cdot k_{i}=n\cdot\left( {2\pi}/{\lambda_{RA}^{TM}} \right)$, $\vec{x}$ and $\boldsymbol{R}_{\boldsymbol{x}}$ are vectors with the same instead of orthogonal direction, so that we can simplify Equation S8:

$\frac{2\pi\text{n}}{\lambda_{\text{RA}}^{\text{TM}}}\text{=-}\frac{2\pi}{\lambda_{\text{RA}}^{\text{TM}}}\cdot\sin\text{θ}_{\text{i}}+\text{p}\cdot\frac{2\pi}{\text{P}_{\text{x}}}$ (S9)

If the $p$ is -1, the calculated $\lambda_{RA}^{TM}$ belongs to a negative number without any meaning, so that the $p$ must be +1, and the $\lambda_{RA}^{TM}$ function is as follows:

$\lambda_{\text{RA}}^{\text{TM}}=\text{P}_{\text{x}}\cdot\left(\text{n}+\sin\text{θ}_{\text{i}}\right)$ (S10)

Similarly, we replace $\theta_{i}$ with $\theta_{SLR}$ and utilize implicit differentiation for Equation S10 to derive the angular sensitivity $S_{\theta}^{TM}$:

$0=\frac{d\left(\text{P}_{\text{x}}\cdot\text{n}+\text{P}_{\text{x}}\cdot\mathrm{s}in\text{θ}_{\text{SLR}}\right)}{d\text{n}}=\text{P}_{\text{x}}+\text{P}_{\text{x}}\cdot\cos\text{θ}_{\text{SLR}}\frac{d\text{θ}_{\text{SLR}}}{d\text{n}}$ (S11)

Therefore, the angular sensitivity $S_{\theta}^{TM}$ is as follows:

 (S12)

**Note 7: Measured and simulated spectra of the TM SLR**

In the case of TM mode, both experimental and simulated SLR wavelengths are redshift with the incident angle increasing from 5° to 25° (Figure S4), meeting the description of Equation 9. This redshift trend is consistent with the SPP but different from the TE SLR totally, which can prove the important influence of polarization in the SLR mechanism.

**
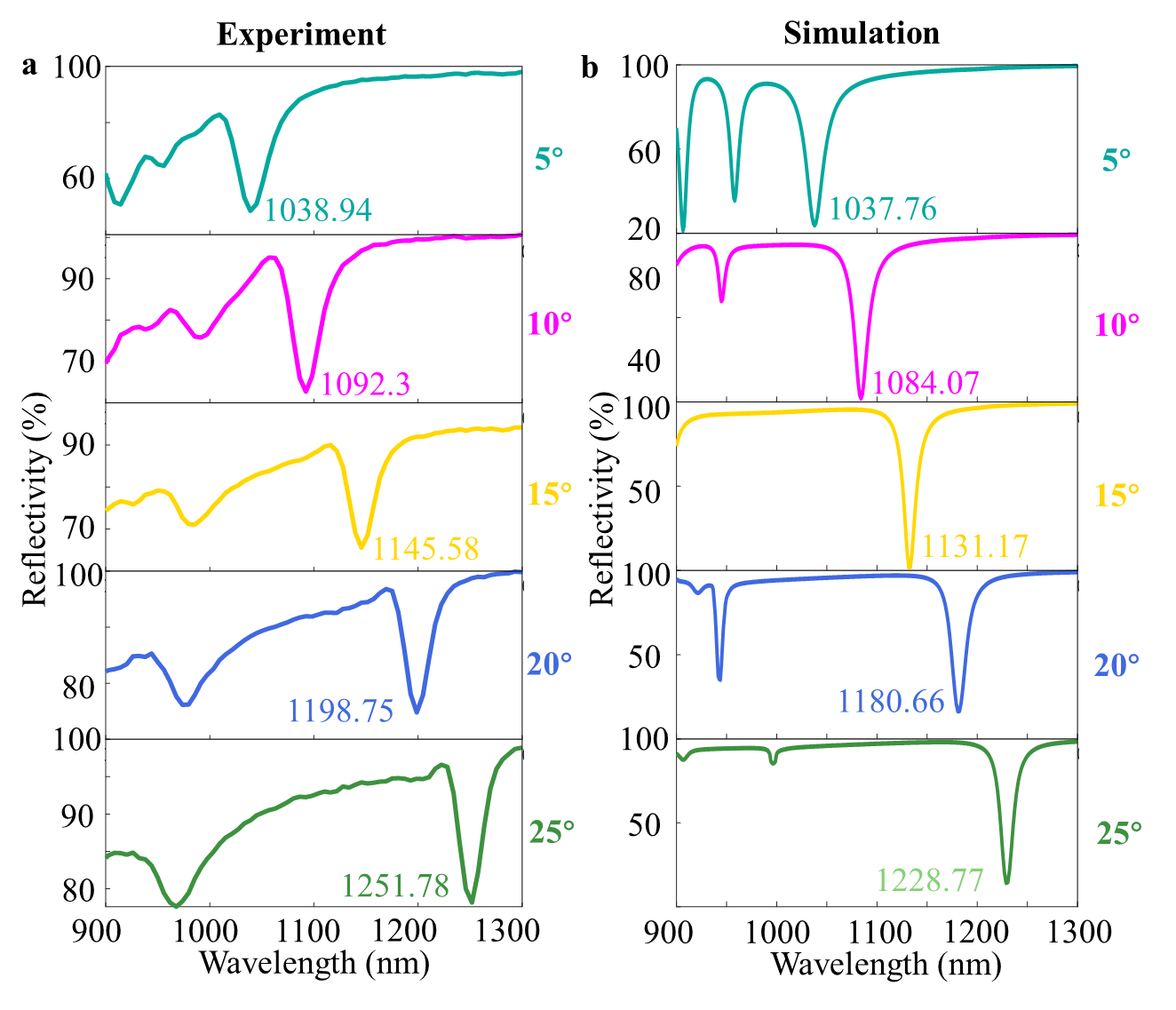
**

**Figure S4.** Measured and simulation spectra of TM incident with different incident angles. **a.** The measured spectra from 5° to 25° at a interval of 5°. **b**. The corresponding numerical simulation results.

**References:**

1. Ashrafi, T., Mohanty, G. Highly sensitive GaN-WS_2_-based surface plasmon resonance sensor: a theoretical approach. *Plasmonics* **17**, 1673-1680 (2022).
2. Hu, C., Surface plasmon resonance sensor based on diffraction grating with high sensitivity and high resolution. *Optik* **122,** 1881-1884 (2011).
3. Kong, L., Lv, J., Gu, Q., Ying, Y., Jiang, X., & Si, G., Sensitivity-enhanced spr sensor based on graphene and subwavelength silver gratings. *Nanomaterials* **10**, 2125 (2020).
4. Cai, D., Lu, Y., Lin, K., Wang, P., & Ming, H. Improving the sensitivity of SPR sensors based on gratings by double-dips method (DDM). *Opt. Express* **16**, 14597-14602 (2008).
5. Mishra, A. K., & Mishra, S. K, Gas sensing in Kretschmann configuration utilizing bi-metallic layer of Rhodium-Silver in visible region. *Sens. Actuators B Chem.* **237**, 969-973 (2016).
6. Su, W., Zheng, G., & Li, X., Design of a highly sensitive surface plasmon resonance sensor using aluminum-based diffraction grating. *Opt. Commun.* 285, 4603-4607 (2012).
7. Ashish, B., & Vipul, R. Sensitivity enhancement of a conventional gold grating assisted surface plasmon resonance sensor by using a bimetallic configuration. *Appl. Opt.* **56**, 9606-9216 (2017).
8. Nur, J. N., Shushama, K. N., Asrafy, F., Hasib, M. H. H., Khan, & M. A. G., Sensitivity enhancement of surface plasmon resonance biosensor using black phosphorus and WSe_2_, International Conference on Robotics, Electrical and Signal Processing Techniques (ICREST 2019), IEEE 576-580 (2019).
9. Wang, H., Tong, C., Guo, X., Li, Z., Shen, J., & Li, C., A high-sensitivity bimetallic grating-coupled surface plasmon resonance sensor based on two-dimensional materials. *Photonics* **10**, 899 (2023).
10. Vibisha, G. A., Daher, M. G., Rahman, S. M. H., Jaroszewicz, Z., Rajesh, K. B., Jha, R., Designing high sensitivity and high figure of merit SPR biosensor using copper and 2D material on CaF_2_ prism, *Results Opt.* **11,** 100407 (2023).
11. Wang, D., Loo, F. C., Cong, H., Lin, W., Kong, S. K., Yam, Y., Chen, S. C., & Ho, H. P., Real-time multi-channel SPR sensing based on DMD-enabled angular interrogation. *Opt. Express* **26**, 24627-24636 (2018).
